# Traumatic Brain Injury and Risk of Malignant Brain Tumors in Civilian Populations

**DOI:** 10.1001/jamanetworkopen.2025.28850

**Published:** 2025-08-25

**Authors:** Sandro Marini, Amr R. Alwakeal, Hunter Mills, Joshua D. Bernstock, Ahmad Mashlah, Muhammad T. Hassan, Jakob Gerstl, Farid Radmanesh, Gundolf Schenk, Sharat Israni, Rachel Grashow, E. Antonio Chiocca, Cathra Halabi, Anthony DiGiorgio, Stephen T. Magill, Geoffrey T. Manley, Saef Izzy, Ross Zafonte

**Affiliations:** 1 Divisions of Stroke, Cerebrovascular, and Critical Care Neurology, Department of Neurology, Brigham and Women’s Hospital, Boston, Massachusetts; 2 Harvard Medical School, Boston, Massachusetts; 3 Department of Neurological Surgery, Northwestern Memorial Hospital, Feinberg School of Medicine, Northwestern University, Chicago, Illinois; 4 Bakar Computational Health Sciences Institute, University of California, San Francisco; 5 Department of Neurosurgery, Brigham and Women’s Hospital, Boston, Massachusetts; 6 Division of Neurocritical Care, Department of Neurosurgery, McGovern Medical School, University of Texas Health Science Center, Houston; 7 Department of Environmental Health, Harvard T. H. Chan School of Public Health, Boston, Massachusetts; 8 The Football Players Health Study at Harvard University, Harvard Medical School, Boston, Massachusetts; 9 Department of Neurology, University of California, San Francisco; 10 Weill Institute for Neurosciences, San Francisco, California; 11 Department of Neurological Surgery, University of California, San Francisco; 12 Department of Physical Medicine and Rehabilitation, Spaulding Rehabilitation Hospital, Harvard Medical School, Boston, Massachusetts; 13 Department of Physical Medicine and Rehabilitation, University of Missouri, Columbia

## Abstract

**Importance:**

A 2024 US study reported that veterans of the Iraq and Afghanistan wars affected by traumatic brain injury (TBI) had an increased risk of developing brain tumors. However, studies among civilians have shown conflicting results and have not replicated the association between TBI and brain tumor risk.

**Objective:**

To examine whether history of TBI in US adult civilians is associated with a risk of subsequent development of malignant brain tumors.

**Design, Setting, and Participants:**

This retrospective cohort study used data from a tertiary academic medical center (Mass General Brigham [MGB]) patient data registry encompassing January 1, 2000, to January 1, 2024. Adult patients (aged >18 years) with mild TBI or moderate to severe TBI (exposure group) were compared with an age- and sex-matched group without a history of TBI (control group). The MGB data were compared across the same time span with data from 2 other tertiary academic medical centers (University of California [UC] Health Data Warehouse and Northwestern Medicine).

**Exposure:**

TBI, which was categorized by severity as mild or moderate to severe.

**Main Outcomes and Measures:**

The primary outcome was development of malignant brain tumor based on *International Classification of Diseases, Ninth Revision* (*ICD-9*) and *Tenth Revision* (*ICD-10*) diagnostic codes in the patient data registry of each cohort. Cox proportional hazards regression analysis was used to determine whether TBI was associated with brain tumor development. The MGB results were then meta-analyzed with the UC Health and Northwestern Medicine data.

**Results:**

The MGB cohort included 151 358 adults: 75 679 control participants (51.8% female; median age, 56 years [IQR, 39-71 years]) and 75 679 civilians with TBI. Of the civilians with TBI (median age, 56 years [IQR, 39-74 years]), 60 735 had mild TBI (54.7% female; median age, 54 years [IQR, 37-73 years]) and 14 944 had moderate to severe TBI (42.1% female; median age, 64 years [IQR, 47-79 years]). The median follow-up duration for the MGB cohort was 7.2 years (IQR, 4.1-10.1 years). Malignant brain tumor prevalence was 0.6% in the moderate to severe TBI group vs 0.4% in the control and mild TBI groups. The moderate to severe TBI group had a higher risk of developing malignant brain tumors (hazard ratio [HR], 1.67 [95% CI, 1.31-2.12]) compared with the mild TBI group (HR, 0.99 [95% CI, 0.83-1.18]). The risk persisted when meta-analyzed with data from the other 2 centers (HR, 1.57 [95% CI, 1.26-1.95]).

**Conclusions and Relevance:**

In this cohort study of US adult civilians, a history of moderate to severe TBI was associated with risk of developing malignant brain tumors. This association was confirmed in a meta-analysis with geographically diverse sites across the US. Additional research is needed to clarify the mechanism through which TBI increases the risk of developing brain tumors.

## Key Points

**Question:**

Is a history of traumatic brain injury (TBI) associated with increased risk of developing a malignant brain tumor among civilian populations?

**Findings:**

In this cohort study including 151 358 US adult civilians, the risk of developing a malignant brain tumor was significantly higher among adults with TBI compared with those without TBI.

**Meaning:**

These findings suggest that additional evaluation and monitoring may be warranted for adult civilians with TBI to detect the potential development of malignant brain tumors.

## Teaser

This cohort study examines whether history of traumatic brain injury (TBI) is associated with risk of subsequent development of malignant brain tumors among US civilian populations.

## Introduction

Traumatic brain injury (TBI) is a leading cause of morbidity and mortality worldwide, with substantial prevalence across the general population.^1,2^ Although the increased mortality risk in individuals with TBI is partially attributable to their heightened risk for chronic diseases,^3^ other potential mechanisms remain underexplored.

Epidemiologic studies have identified various potential risk factors for brain tumors, with ionizing radiation being the only consistently validated association.^4^ Experimental studies in mice and humans demonstrate that structural brain injury induces reactive astrocytes, triggering localized inflammation, astrogliosis, and glial scar formation.^5,6,7^ These findings raise the possibility that TBI may act as a risk factor for brain tumor development.

Supporting this hypothesis, research in military populations has indicated an association between moderate, severe, and penetrating TBI and an increased risk of malignant brain tumors.^8^ However, the unique characteristics of military populations, such as exposure to combat-related stressors and exposure to peculiar toxins via chemicals or burn pits, complicate the generalizability of these findings to civilian populations. Nationwide cohort studies in civilian populations in Denmark and Sweden have shown no association between TBI and malignant astrocytic tumors.^9,10^ Furthermore, a 2022 systematic review examining the association between TBI and subsequent development of malignant brain tumors highlighted methodologic limitations, including differences in data collection, substantial recall bias, and selection bias.^11^ Consequently, the association between TBI in adults and development of malignant brain tumors remains inconsistent and inconclusive.

Given the high prevalence of TBI and the potential consequence of early tumor detection on health outcomes, it is imperative to clarify whether TBI is associated with an elevated risk of developing malignant brain tumors. In this study, we present findings from 3 longitudinal cohort studies spanning 20 years to assess the risk and latency of brain tumor diagnoses following TBI among US adult civilians.

## Methods

The Institutional Review Boards of Mass General Brigham (MGB), the University of California (UC), and Northwestern University waived formal review and informed consent for this cohort study because the data were deidentified and the study did not meet the definition of human participant research. This study followed the Strengthening the Reporting of Observational Studies in Epidemiology (STROBE) reporting guideline.

### Patient Selection

Our primary analysis used data from MGB, a tertiary trauma center in Boston, Massachusetts. We then used data from other trauma centers across the US as follows: (1) trauma centers affiliated with Northwestern Medicine in Chicago, Illinois; and (2) the UC Health Data Warehouse, which harmonizes electronic health records (EHRs) from 6 UC sites (Davis, Irvine, Los Angeles, Riverside, San Diego, and San Francisco). MGB used the Research Patient Data Registry, which is a web-based application that allows query of a prospectively collected, centralized clinical data registry encompassing inpatient, outpatient, and emergency department diagnoses. The UC Health system used the UC Data Discovery Portal, an Observational Medical Outcomes Partnership database unifying fully structured EHR data from multiple health care systems across California.^12^ Northwestern Medicine used its Enterprise Data Warehouse, a centralized repository that integrates EHRs, pathology reports, and diagnostic data from inpatient, outpatient, and emergency department encounters across Northwestern Memorial Hospital and its affiliated institutions. To define exposure and outcome, all 3 centers used similar methodology, which has been widely used in the literature.^13,14,15^ We defined TBI and malignant brain tumor using the US Centers for Disease Control and Prevention criteria and extracted participants using validated *International Classification of Diseases*, *Ninth Revision* (*ICD-9*) and *Tenth Revision* (*ICD-10*) diagnostic codes. We used the same codes and definitions for a malignant brain tumor as those described previously.^8^ We divided patients with TBI into mild TBI and moderate to severe TBI groups based on *ICD-9* codes. The UC Health system used the Abbreviated Injury Scale for head and neck.

All 3 centers performed a retrospective cohort study from January 1, 2000, to January 1, 2024. We included patients older than 18 years who had at least 2 inpatient or outpatient visits for TBI and a minimum of 2 inpatient or outpatient follow-up visits (exposure group). The index date was first TBI diagnosis. We selected at least a 1-year lag to avoid identifying tumors that were undiagnosed at the time of the TBI. All centers identified control participants (unexposed to TBI) by selecting patients propensity score matched to age, sex, and race and ethnicity distribution as their TBI group (control group). For the MGB dataset, we conducted a sensitivity analysis that included a second group of patients matched specifically on the characteristics of the moderate to severe TBI group. For the control group only, the index date was randomly chosen to minimize bias inherent in data from national neurosurgical referral centers. We included control participants who had at least 2 inpatient or outpatient visits before the index date. For both patients with TBI and control participants, we excluded those with a history of TBI and any tumor or head and neck radiation exposure. Participant demographics were those at the time of the index date. Race and ethnicity are important covariates in the epidemiology of TBI and access to health resources. These data were collected from medical records or patient self-reports and were categorized as follows: American Indian or Alaska Native, Asian, Black, Hispanic, White, other race or ethnicity (defined as Middle Eastern or Native Hawaiian or Other Pacific Islander and included those who selected multiple races or ethnicities), and unknown race or ethnicity.^3,13,15^

### Statistical Analysis

Counts with proportions or medians with IQRs are reported for descriptive analyses, as appropriate. We used the χ^2^ test to estimate differences across baseline characteristics among groups. To assess associations between TBI and brain tumor risk, we used Kaplan-Meier plots with log-rank tests to compare cumulative incidence rates between the exposure and control groups. We used Cox proportional hazards regression models to adjust for age, sex, and race and ethnicity and assessed the proportional hazards assumption using Schoenfeld residuals. A second model included only patients with moderate to severe TBI and their corresponding matched control participants. We censored patients and control participants at the time of brain tumor diagnosis, death, or the last available encounter, whichever came first. The matching of control participants was performed via propensity score methodology using the algorithm method that achieved the smallest absolute within-pair differences for each covariate.^16^ Initially, we performed the analysis in the MGB cohort. To further confirm the results, we performed a meta-analysis with data from the other 2 US centers (UC Health Data Warehouse and Northwestern Medicine). We meta-analyzed data for TBI across centers using the DerSimonian and Laird random-effects model with weights calculated by the inverse variance method.^17^ We quantified the associations with outcomes of interest using hazard ratios (HRs) with 95% CIs. We assessed heterogeneity by *I*^2^ and χ^2^ statistics and visually through inspection of the forest plot. We considered 2-sided *P* < .05 statistically significant. All analyses were performed using RStudio, version 4.0.2 (R Project for Statistical Computing).^18^

## Results

**Figure 1.**
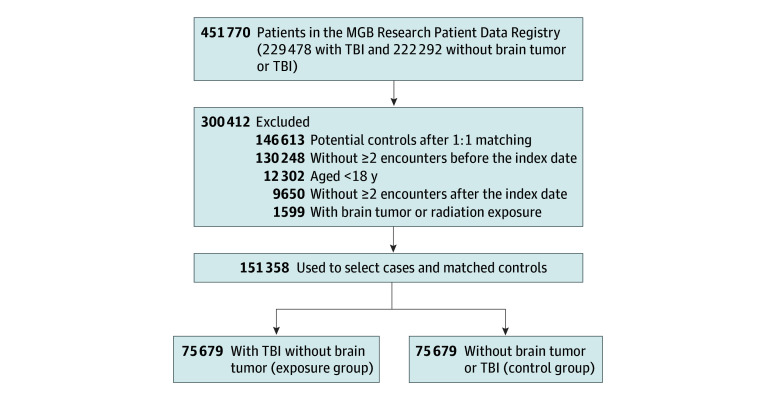
Flowchart of the Mass General Brigham (MGB) Cohort TBI indicates traumatic brain injury.

Of the available individuals in the MGB data registry, we included 151 358 adults: 75 679 patients with TBI and 75 679 matched control participants (Figure 1). The control group was matched to the exposure group for age, sex, race and ethnicity, and number of follow-up encounters (eTable 1 in Supplement 1). Both sexes were evenly presented across groups, with 51.8% female and 48.2% male participants in the control group and 52.2% female and 47.8% male participants in the exposure group. The median age at the index date was 56 years (IQR, 39-71 years) for control participants and 56 years (IQR, 39-74 years) for patients with TBI (54 years [IQR, 37-73 years] for patients with mild TBI and 64 years [IQR, 47-79 years] for patients with moderate to severe TBI). Follow-up duration was slightly more extended for control participants (median, 4.9 years [IQR, 2.1-9.0 years]) compared with patients with TBI (median, 4.6 years [IQR,1.4-9.0]). Race and ethnicity was similarly represented among control participants and patients with TBI (American Indian or Alaska Native, 0.2% and 0.3%; Asian, 2.0% and 2.2%; Black, 7.0% and 7.0%; Hispanic, 3.4% and 3.4%; White, 79.4% and 79.2%; and other or unknown race or ethnicity, 7.9% and 8.0%) (*P* = .13). The number of encounters was significantly higher in patients with TBI compared with control participants before the index date (median, 20 [IQR, 7-58] vs 10 [4-25]) but similar after the index date (median, 32 [IQR, 10-94] vs 34 [13-74]).

Table 1 presents the descriptive characteristics of the MGB cohort across strata of TBI severity. Of all 75 679 patients with TBI, 60 735 (80.3%) had mild TBI and 14 944 (19.7%) had moderate to severe TBI. The median follow-up duration for the MGB cohort was 7.2 (IQR, 4.1-10.1) years. Compared with the control group, the moderate to severe TBI group had more males (57.9% vs 48.2%). The moderate to severe TBI group was older (median age, 64 years [IQR, 47-79 years]) compared with the mild TBI group (median age, 54 years [IQR, 37-73 years]) and the control group (median age, 56 years [IQR, 39-71 years]). The moderate to severe TBI group had a higher percentage of White patients (82.7%) compared with the mild TBI group (78.3%) and the control group (79.4%) (overall *P* < .001). Follow-up duration was shorter for the moderate to severe TBI group (median, 3.2 years [IQR, 0.5-8.1 years]) compared with the control group (median, 4.9 years [IQR, 2.1-9.0 years]) and the mild TBI group (median, 5.0 years [IQR, 1.6-9.2 years]). The median number of encounters before the index date was 10 (IQR, 4-25) for the control group, 21 (IQR, 7-61) for the mild TBI group, and 15 (IQR, 5-47) for the moderate to severe TBI group. eTable 2 in Supplement 1 describes 14 944 control participants in the MGB cohort matched specifically to the 14 944 patients in the moderate to severe TBI group. Similarly to the overall groups, the moderate to severe TBI group had shorter follow-up but a higher number of encounters (eTable 2 in Supplement 1).

**Table 1.** Baseline Characteristics of Patients With TBI and Control Participants in the Mass General Brigham Cohort (N = 151 358)

Characteristic	Control group (n = 75 679)	Exposure group (n = 75 679)		*P* value
		Mild TBI (n = 60 735)	Moderate to severe TBI (n = 14 944)	
Sex, No (%)				
Female	39 183 (51.8)	33 217 (54.7)	6293 (42.1)	<.001
Male	36 484 (48.2)	27 503 (45.3)	8650 (57.9)	
Unknown	12 (<0.01)	15 (<0.01)	<10 (<0.01)^a^	
Age at TBI, median (IQR), y	56 (39-71)	54 (37-73)	64 (47-79)	<.001
Race and ethnicity, No (%)				
American Indian or Alaska Native	179 (0.2)	167 (0.3)	36 (0.2)	<.001
Asian	1523 (2.0)	1321 (2.2)	336 (2.2)	
Black	5328 (7.0)	4584 (7.5)	742 (5.0)	
Hispanic	2597 (3.4)	2158 (3.6)	401 (2.7)	
White	60 110 (79.4)	47 543 (78.3)	12 357 (82.7)	
Other or unknown^b^	5942 (7.9)	4962 (8.2)	1072 (7.2)	
Follow-up duration, median (IQR), y	4.9 (2.1-9.0)	5.0 (1.6-9.2)	3.2 (0.5-8.1)	<.001
No. of encounters after the index date, median (IQR),	34 (13-74)	33 (10-96)	31 (11-85)	<.001
No. of encounters before the index date, median (IQR)	10 (4-25)	21 (7-61)	15 (5-47)	<.001
Malignant brain tumor incidence, No (%)	314 (0.4)	222 (0.4)	87 (0.6)	.001

Abbreviation: TBI, traumatic brain injury.

^a^ Cell sizes less than 10 are redacted in accordance with data privacy and reporting standards.

^b^ Defined as Middle Eastern or Native Hawaiian or Other Pacific Islander and included those who selected multiple races or ethnicities.

**Figure 2.**
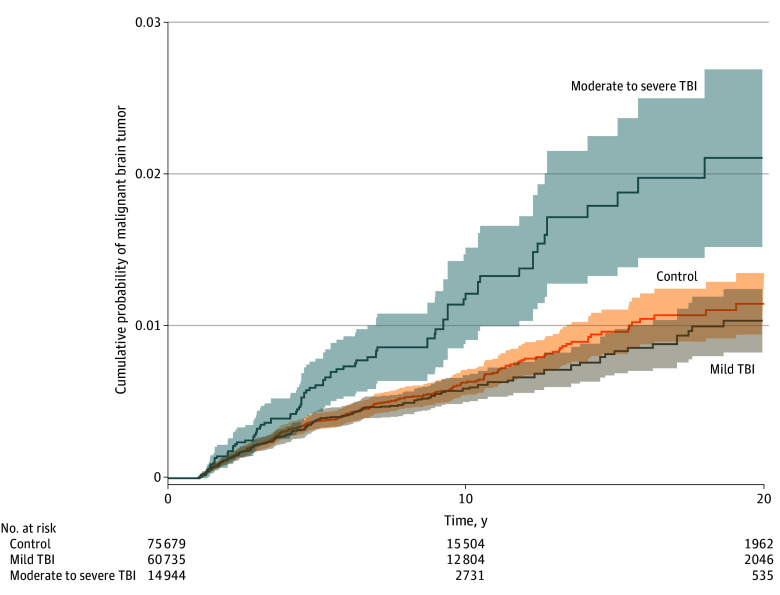
Kaplan-Meier Curves for Malignant Brain Tumor Incidence Across Patients With Traumatic Brain Injury (TBI) and Control Participants in the Mass General Brigham Cohort The y-axis is restricted to 97% to 100% for visibility. Shaded areas indicate 95% CIs.

In the MGB cohort, malignant brain tumor incidence was higher in the moderate to severe TBI group (87 [0.6%]) compared with the control group (314 [0.4%]) and the mild TBI group (222 [0.4%]) (*P* = .001) (Figure 2 and Table 1). The moderate to severe TBI group had a higher percentage of malignant brain tumors (87 [0.6%]) compared with the matched control group (64 [0.4%]) with similar age, sex, and race and ethnicity (*P* = .003) (eTable 2 in Supplement 1). Moderate to severe TBI was associated with malignant brain tumors in the Cox proportional hazards regression analysis (HR, 1.67 [95% CI, 1.31-2.12]; *P* < .001), independently of age, sex, and race and ethnicity (Table 2).

**Table 2.** Multivariable Cox Proportional Hazards Regression Analysis for the Outcome of Malignant Brain Tumors in the Mass General Brigham Cohort

Group	Hazard ratio (95% CI)	*P* value
TBI		
Mild	0.99 (0.83-1.18)	.91
Moderate to severe	1.67 (1.31-2.12)	<.001
Male sex	1.12 (0.95-1.31)	.20
Age	1.02 (1.02-1.02)	<.001
Race and ethnicity		
American Indian or Alaska Native	0 (NA)^a^	>.99
Asian	0.48 (0.21-1.07)	.07
Black	0.73 (0.50-1.05)	.09
Hispanic	0.97 (0.59-1.61)	.91
White	[Reference]	NA
Other or unknown^b^	1.03 (0.77-1.40)	.85

Abbreviations: NA, not applicable; TBI, traumatic brain injury.

^a^ Statistical comparison was not performed owing to the small sample size.

^b^ Defined as Middle Eastern or Native Hawaiian or Other Pacific Islander and included those who selected multiple races or ethnicities.

Even in the model comparing control participants specifically matched to patients with moderate to severe TBI, the presence of trauma was independently associated with malignant brain tumors (HR, 1.47 [95% CI, 1.06-2.04]; *P* = .02) (eTable 3 in Supplement 1). Mild TBI was not associated with brain tumors (HR, 0.99 [95% CI, 0.83-1.18]; *P* = .91). Schoenfeld residuals of both Cox proportional hazards regression analyses were consistent with the proportional hazards assumption (*P* = .25).

There were 39 403 patients with TBI and 39 403 matched control participants in the UC Health cohort (eTable 4 in Supplement 1). Compared with males, females represented 54.7% vs 45.3% of the control group, 57.2% vs 42.3% of the mild TBI group, and 41.5% vs 58.5% of the moderate to severe TBI group. Sex and age distributions were similar to the MGB cohort, with a higher percentage of older males in the moderate to severe TBI group. Compared with the MGB cohort, follow-up duration was similar but with a higher number of encounters before the index date and fewer after the index date (eTable 4 in Supplement 1).

The Northwestern Medicine cohort contributed 16 222 patients with TBI and 16 222 matched control participants (eTable 5 in Supplement 1). The cohort had overall fewer male participants (62.8% female vs 37.2% male in the control group, 65.8% vs 34.2% in the mild TBI group, and 46.6% vs 53.4% in the moderate to severe TBI group). The number of encounters (both pre and post index) was overall higher compared with the MGB cohort (eTable 5 in Supplement 1).

**Figure 3.**
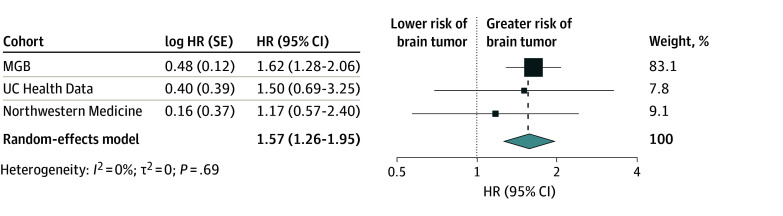
Forest Plot of the Random-Effects Model With Inverse Variance Method Meta-Analysis of Malignant Brain Tumor Risk Among Participants With Moderate to Severe Traumatic Brain Injury (TBI) Across 3 Cohorts The different box sizes reflect the weight of each study in the meta-analysis. HR indicates hazard ratio; MGB, Mass General Brigham; UC, University of California.

For all 3 cohorts, White was the most prevalent race. Malignant brain tumor incidence was similar across the control, mild TBI, and moderate to severe TBI groups for both the UC Health cohort (38 [0.1%], 50 [0.2%], and <10 [0.1%]; *P* = .11) and the Northwestern Medicine cohort (13 [<0.1%], 13 [<0.1%], and <10 [0.2%]; *P* = .40). However, when combined in the random-effects model meta-analysis, moderate to severe TBI was associated with an increase in subsequent development of malignant brain tumors by approximately 50% (HR, 1.57 [95% CI, 1.26-1.95]) (Figure 3).

## Discussion

In this cohort study, we found that moderate to severe TBI, but not mild TBI, was associated with increased risk of any type of malignant brain tumor. Our study included a large number of civilian patients and benefited from a combination of different TBI populations across the US, adding to the research on whether adults with TBI are at increased risk for developing post-TBI brain tumors.

The findings of previous studies evaluating the association between TBI and brain tumor development have been inconsistent.^11^ Chen et al^19^ found a significantly higher risk of brain tumor development in a cohort of Taiwanese patients hospitalized with TBI compared with control participants. A few study characteristics, including the short follow-up period, the small number of patients with severe TBI and brain tumors, and the limited geographic region and demographic diversity, may limit the generalizability of their findings. Conversely, investigators conducted 3 cohort studies in the Northern European population and did not find any significantly increased risk of brain tumor development for patients with TBI.^9,10,20^ Our study differs from the current literature in that our cohort includes more diverse geographic and racial patient populations, a wider participant age range, and a large number of participants with severe TBI. Additionally, we applied a control matching system and adjusted for number of encounters to mitigate ascertainment bias (in the aforementioned studies) related to more follow-up visits that patients with TBI may receive for their injuries. Our detailed TBI definition, in line with previous studies,^13,15^ and higher number of participants compensate for the limited power that these previous studies may have had. Our findings are also supported by a 2024 study carried out in the specific population of US veterans,^8^ in which veterans experienced almost twice the risk for brain tumor for nonpenetrating TBI and more than 3 times the risk for penetrating TBI compared with control participants. This correlation may be limited to the military population (skewed toward young age and male sex) or driven by confounders not accounted for, such as potential toxic exposures otherwise uncommon in civilians. Our results contribute to the literature in that we studied a civilian population and identified an association between moderate to severe TBI and brain tumor risk, which may contribute to increased mortality seen in patients with TBI.^21^

Although the UC Health and Northwestern Medicine data showed a nonsignificant increase in brain tumor risk in patients with TBI and significant results were seen in the dataset that leveraged the larger data (especially the higher number of patients with severe TBI), all 3 studies analyzed here had concordant effect sizes. This finding is supportive of the high power that epidemiology studies need to assess for this relatively rare outcome, as also shown by the aforementioned 2024 veterans study,^8^ which enrolled more than a million individuals, and the inconsistency between results of previous studies.^11^ Furthermore, the maintenance of statistical significance when data were pooled may argue against a potential spurious effect coming from one center.

The mechanism through which TBI increases the risk of brain tumor development is not understood. A variety of potential pathologic pathways may link the 2 phenomena. TBI triggers metabolic derangements, such as in glucose metabolism, which can increase free radicals.^22^ TBI also causes acute and chronic neuroinflammatory responses, and a growing body of research is linking inflammatory mediators to cell proliferation, migration, and angiogenesis.^23,24^ Finally, TBI leads to a heterogeneous astrocyte response including astrocyte hypertrophy and increased proliferative capacity.^25^ Such hypothesized mechanisms could partially account for the link between TBI and brain tumor development identified in this study and motivate additional research.

### Limitations

Our study has limitations. We were unable to distinguish the type of malignant tumor associated with TBI. Knowing the histologic cancer type could advance our current knowledge of the pathologic cascade that leads from TBI to neoplasia. We did not adjust for the number of radiation exposures that case patients and control participants are exposed to from imaging studies. We were unable to stratify our cohort by patient characteristics (eg, socioeconomic status) or lifestyle-related factors (eg, smoking or alcohol consumption) due to lack of data. However, the only established environmental risk factor for developing brain tumors is ionizing radiation; thus, including such additional variables may not have yielded usable insight.^26^ We also could not identify patients who experienced multiple TBIs and cannot determine whether the association seen in this study resulted from repeated TBIs. Future research should explore whether repeated TBIs further enhance brain tumor risk. Finally, we could not investigate the association between TBI with penetration injury and brain tumor development due to a very limited number of case patients.

## Conclusions

In this cohort study of civilians, a history of moderate to severe TBI was associated with subsequent risk of developing malignant brain tumors. This association was confirmed in a meta-analysis with geographically diverse sites across the US. This analysis leveraged, to our knowledge, the largest cohort of nonveterans with TBI, and identified an association between TBI and malignant brain tumor development in adults that may have been gone undetected in previous smaller studies. These findings prompt studies aimed at answering whether specific screening programs for brain tumors should be implemented following TBI. Future studies should clarify whether TBI increases risk directly, indirectly, or via other spurious confounding associations.

## Notes

Supplement 1. **eTable 1.** Baseline Characteristics of Patients With TBI and Matched Control Participants in the Mass General Brigham Cohort **eTable 2.** Baseline Characteristics of Patients With Moderate to Severe TBI and Corresponding Matched Control Participants in the Mass General Brigham Cohort **eTable 3.** Multivariable Cox Regression Analysis for Malignant Brain Tumor Outcomes in Patients With Moderate to Severe TBI and Corresponding Matched Control Participants in the Mass General Brigham Cohort **eTable 4.** Baseline Characteristics of the University of California Health Cohort **eTable 5.** Baseline Characteristics of the Northwestern Medicine Cohort

Supplement 2.
Data Sharing Statement

## References

[1] Dewan MC, Rattani A, Gupta S, . Estimating the global incidence of traumatic brain injury. J Neurosurg. 2018;130(4):1080-1097. doi:10.3171/2017.10.JNS17352 29701556

[2] Hyder AA, Wunderlich CA, Puvanachandra P, Gururaj G, Kobusingye OC. The impact of traumatic brain injuries: a global perspective. NeuroRehabilitation. 2007;22(5):341-353. doi:10.3233/NRE-2007-22502 18162698

[3] Izzy S, Grashow R, Radmanesh F, . Long-term risk of cardiovascular disease after traumatic brain injury: screening and prevention. Lancet Neurol. 2023;22(10):959-970. doi:10.1016/S1474-4422(23)00241-7 37739576 PMC10863697

[4] Ostrom QT, Adel Fahmideh M, Cote DJ, . Risk factors for childhood and adult primary brain tumors. Neuro Oncol. 2019;21(11):1357-1375. doi:10.1093/neuonc/noz123 31301133 PMC6827837

[5] Michinaga S, Koyama Y. Pathophysiological responses and roles of astrocytes in traumatic brain injury. Int J Mol Sci. 2021;22(12):22. doi:10.3390/ijms22126418 34203960 PMC8232783

[6] Jassam YN, Izzy S, Whalen M, McGavern DB, El Khoury J. Neuroimmunology of traumatic brain injury: time for a paradigm shift. Neuron. 2017;95(6):1246-1265. doi:10.1016/j.neuron.2017.07.010 28910616 PMC5678753

[7] Simińska D, Kojder K, Jeżewski D, . The pathophysiology of post-traumatic glioma. Int J Mol Sci. 2018;19(8):19. doi:10.3390/ijms19082445 30126222 PMC6121393

[8] Stewart IJ, Howard JT, Poltavskiy E, . Traumatic brain injury and subsequent risk of brain cancer in US veterans of the Iraq and Afghanistan wars. JAMA Netw Open. 2024;7(2):e2354588. doi:10.1001/jamanetworkopen.2023.54588 38358743 PMC10870183

[9] Munch TN, Gørtz S, Wohlfahrt J, Melbye M. The long-term risk of malignant astrocytic tumors after structural brain injury—a nationwide cohort study. Neuro Oncol. 2015;17(5):718-724. doi:10.1093/neuonc/nou312 25416827 PMC4482857

[10] Nygren C, Adami J, Ye W, . Primary brain tumors following traumatic brain injury—a population-based cohort study in Sweden. Cancer Causes Control. 2001;12(8):733-737. doi:10.1023/A:1011227617256 11562113

[11] Shah DS, Sanan A, Morell AA, . Traumatic brain injury and subsequent brain tumor development: a systematic review of the literature. Neurosurg Rev. 2022;45(5):3003-3018. doi:10.1007/s10143-022-01819-y 35641842

[12] Halabi C, Izzy S, DiGiorgio AM, . Traumatic brain injury and risk of incident comorbidities. JAMA Netw Open. 2024;7(12):e2450499. doi:10.1001/jamanetworkopen.2024.50499 39666337 PMC11638795

[13] Izzy S, Chen PM, Tahir Z, . Association of traumatic brain injury with the risk of developing chronic cardiovascular, endocrine, neurological, and psychiatric disorders. JAMA Netw Open. 2022;5(4):e229478. doi:10.1001/jamanetworkopen.2022.9478 35482306 PMC9051987

[14] Izzy S, Tahir Z, Grashow R, . Concussion and risk of chronic medical and behavioral health comorbidities. J Neurotrauma. 2021;38(13):1834-1841. doi:10.1089/neu.2020.7484 33451255 PMC8219193

[15] Stopa BM, Tahir Z, Mezzalira E, . The impact of age and severity on dementia after traumatic brain injury: a comparison study. Neurosurgery. 2021;89(5):810-818. doi:10.1093/neuros/nyab297 34392366

[16] Ho DE, Imai K, King G, Stuart EA. Matching as nonparametric preprocessing for reducing model dependence in parametric causal inference. Polit Anal. 2007;15:199-236. doi:10.1093/pan/mpl013

[17] DerSimonian R, Laird N. Meta-analysis in clinical trials. Control Clin Trials. 1986;7(3):177-188. doi:10.1016/0197-2456(86)90046-2 3802833

[18] R Core Team. R: A Language and Environment for Statistical Computing. R Foundation for Statistical Computing; 2013.

[19] Chen YH, Keller JJ, Kang JH, Lin HC. Association between traumatic brain injury and the subsequent risk of brain cancer. J Neurotrauma. 2012;29(7):1328-1333. doi:10.1089/neu.2011.2235 22320191

[20] Inskip PD, Mellemkjaer L, Gridley G, Olsen JH. Incidence of intracranial tumors following hospitalization for head injuries (Denmark). Cancer Causes Control. 1998;9(1):109-116. doi:10.1023/A:1008861722901 9486470

[21] Harrison-Felix C, Pretz C, Hammond FM, . Life expectancy after inpatient rehabilitation for traumatic brain injury in the United States. J Neurotrauma. 2015;32(23):1893-1901. doi:10.1089/neu.2014.3353 25057965 PMC6082166

[22] Demers-Marcil S, Coles JP. Cerebral metabolic derangements following traumatic brain injury. Curr Opin Anaesthesiol. 2022;35(5):562-569. doi:10.1097/ACO.0000000000001183 35943124 PMC9594147

[23] Groblewska M, Litman-Zawadzka A, Mroczko B. The role of selected chemokines and their receptors in the development of gliomas. Int J Mol Sci. 2020;21(10):21. doi:10.3390/ijms21103704 32456359 PMC7279280

[24] Perng P, Lim M. Immunosuppressive mechanisms of malignant gliomas: parallels at non-CNS sites. Front Oncol. 2015;5:153. doi:10.3389/fonc.2015.00153 26217588 PMC4492080

[25] Muñoz-Ballester C, Robel S. Astrocyte-mediated mechanisms contribute to traumatic brain injury pathology. WIREs Mech Dis. 2023;15(5):e1622. doi:10.1002/wsbm.1622 37332001 PMC10526985

[26] Michaud DBT. Risk factors for brain tumors. UpToDate. 2024. Accessed December 23, 2024. https://www.uptodate.com/contents/risk-factors-for-brain-tumors

