# Supplementary material for: Traumatic Brain Injury and Risk of Malignant Brain Tumors in Civilian Populations
Source: JAMA Netw Open. 2025 Aug 25;8(8):e2528850. doi: 10.1001/jamanetworkopen.2025.28850 (PMC12379090; doi:10.1001/jamanetworkopen.2025.28850)
Supplement: Supplement 1. — eTable 1. Baseline Characteristics of Patients With TBI and Matched Control Participants in the Mass General Brigham Cohort eTable 2. Baseline Characteristics of Patients With Moderate to Severe TBI and Corresponding Matched Control Participants in the Mass General Brigham Cohort eTable 3. Multivariable Cox Regression Analysis for Malignant Brain Tumor Outcomes in Patients With Moderate to Severe TBI and Corresponding Matched Control Participants in the Mass General Brigham Cohort eTable 4. Baseline Characteristics of the University of California Health Cohort eTable 5. Baseline Characteristics of the Northwestern Medicine Cohort [file jamanetwopen-e2528850-s001.pdf]

Supplemental Online Content

Marini S, Alwakeal AR, Mills H, et al. Traumatic brain injury and risk of malignant brain tumors in civilian populations. *JAMA Netw Open*. 2025;8(8):e2528850. doi:10.1001/jamanetworkopen.2025.28850

eTable 1. Baseline Characteristics of Patients With TBI and Matched Control Participants in the Mass General Brigham Cohort

eTable 2. Baseline Characteristics of Patients With Moderate to Severe TBI and Corresponding Matched Control Participants in the Mass General Brigham Cohort

eTable 3. Multivariable Cox Regression Analysis for Malignant Brain Tumor Outcomes in Patients With Moderate to Severe TBI and Corresponding Matched Control Participants in the Mass General Brigham Cohort

eTable 4. Baseline Characteristics of the University of California Health Cohort

eTable 5. Baseline Characteristics of the Northwestern Medicine Cohort

This supplemental material has been provided by the authors to give readers additional information about their work.

© 2025 Marini S et al. *JAMA Network Open*.

eTable 1. Baseline Characteristics of Patients With TBI and Matched Control Participants in the Mass General Brigham Cohort

**Characteristic**		**Control group N=75,679**	**TBI N=75,679**	**p-value**
Severity	Mild		60,735 (80.3%)	
	Moderate/Severe		14,944 (19.7%)	
Sex	Female	39,183 (51.8%)	39,510 (52.2%)	0.20
	Male	36,484 (48.2%)	36,153 (47.8%)	
	Unknown	12 (<0.01%)	16 (<0.01%)	
Age at TBI, years (median, IQR)		56 (39-71)	56 (39, 74)	<0.01
Race and Ethnicity	American Indian or Alaska Native	179 (0.2%)	203 (0.3%)	0.13
	Asian	1,523 (2.0%)	1,657 (2.2%)	
	Black	5,328 (7.0%)	5,326 (7.0%)	
	Hispanic	2,597 (3.4%)	2,559 (3.4%)	
	White	60,110 (79.4%)	59,900 (79.2%)	
	Other or unknown race or ethnicity**	5,942 (7.9%)	6,034 (8.0%)	
Follow up in years (median, IQR)		4.9 (2.1-9.0)	4.6 (1.4, 9.0)	<0.01
Numbers of encounters after index date (IQR)		34 (13-74)	32 (10, 94)	0.23
Numbers of encounters before index date (IQR)		10 (4-25)	20 (7-58)	<0.01
Malignant Brain Tumor incidence		314 (0.4%)	309 (0.4%)	0.82

IQR: interquartile range; TBI: Traumatic Brain Injury. *Cell sizes less than 10 are redacted in accordance with data privacy and reporting standards. **Defined as Asian Pacific Islander, Hawaiian, or Middle Eastern, and included those who selected multiple races or ethnicities.

© 2025 Marini S et al. *JAMA Network Open*.

eTable 2. Baseline Characteristics of Patients With Moderate to Severe TBI and Corresponding Matched Control Participants in the Mass General Brigham Cohort

Characteristic		Control group N = 14,944	Moderate to Severe N = 14,944	p-value
Sex	Female	6,156 (41%)	6,293 (42.1%)	0.11
	Male	8,788 (59%)	8,650 (57.9%)	
	Unknown	0 (0%)	<10 (<0.01%)	
Age at TBI, years (median, IQR)		64 (47-77)	64 (47-79)	0.02
Race and Ethnicity	American Indian or Alaska Native	38 (0.3%)	36 (0.2%)	
	Asian	335 (2.2%)	336 (2.2%)	
	Black	729 (4.9%)	742 (5.0%)	
	Hispanic	420 (2.8%)	401 (2.7%)	
	White	12,352 (83%)	12,357 (82.7%)	
	Other or unknown race or ethnicity**	1,070 (7.2%)	1,072 (7.2%)	
Follow up in years (median, IQR)		4.2 (1.5-7.7)	3.2 (0.5-8.1)	<0.001
Numbers of encounters after index date (IQR)		30 (12-67)	31 (11-85)	<0.001
Numbers of encounters before index date (IQR)		9 (4-24)	15 (5-47)	<0.001
Malignant Brain Tumor incidence		64 (0.4%)	87 (0.6%)	0.003

IQR: interquartile range; TBI: Traumatic Brain Injury. *Cell sizes less than 10 are redacted in accordance with data privacy and reporting standards. **Defined as Asian Pacific Islander, Hawaiian, or Middle Eastern, and included those who selected multiple races or ethnicities.

© 2025 Marini S et al. *JAMA Network Open*.

eTable 3. Multivariable Cox Regression Analysis for Malignant Brain Tumor Outcomes in Patients With Moderate to Severe TBI and Corresponding Matched Control Participants in the Mass General Brigham Cohort

Group		HR	95%CI	p-value
Moderate to Severe TBI		1.47	1.06-2.04	0.02
Male sex		1.15	0.87-1.53	0.33
Age		1.02	1.01-1.02	<0.001
Race/ethnicity	American Indian or Alaska Native	0.00	n.a	>0.99
	Asian	0.22	0.03-1.60	0.14
	Black	0.70	0.33-1.49	0.40
	Hispanic	1.02	0.42-2.49	>0.9
	White	ref		
	Other or unknown race or ethnicity**	1.19	0.71-1.99	0.51

HR: Hazard Ratio; CI: Confidence Intervals; TBI: Traumatic Brain Injury. *Cell sizes less than 10 are redacted in accordance with data privacy and reporting standards. **Defined as Asian Pacific Islander, Hawaiian, or Middle Eastern, and included those who selected multiple races or ethnicities.

© 2025 Marini S et al. *JAMA Network Open*.

eTable 4. Baseline Characteristics of the University of California Health Cohort

Characteristic		Control group N=39,403	Mild TBI N=32,982	Moderate to Severe TBI N=6,421	p-value
Sex	Female	21548 (54.7%)	18881 (57.2%)	2667 (41.5%)	<0.001
	Male	17855 (45.3%)	14101 (42.3%)	3754 (58.5%)	
Age at TBI, years (median, IQR)		59 (42-72)	58 (39-74)	63 (48-76)	<0.001
Race and ethnicity	American Indian or Alaska Native	130 (0.3%)	110 (0.3%)	20 (0.3%)	<0.001
	Asian	3624 (9.2%)	2952 (9.0%)	672 (10.5%)	
	Black	3135 (8.0%)	2605 (7.9%)	530 (8.3%)	
	Hispanic	6299 (16.0%)	5147 (15.6%)	1152 (17.9%)	
	White	22978 (58.3%)	19368 (58.7%)	3610 (56.2%)	
	Other or unknown race or ethnicity**	3237 (8.2%)	2800 (8.5%)	437 (6.8%)	
Follow up in years (median, IQR)		4.1 (1.8-6.7)	4.4 (2.2-6.6)	3.5 (0.9-5.7)	<0.001
Numbers of encounters after index date (IQR)		7 (3-17)	23 (7-63)	16 (5-55)	<0.001
Numbers of encounters before index date (IQR)		22 (9-57)	42 (13-113)	27 (8-85)	<0.001
Malignant Brain Tumor incidence		38 (0.1%)	50 (0.2 %)	<10* (0.1%)	0.11
Location	UCD	9547 (24.2%)	7713 (23.4%)	1834 (28.6%)	
	UCI	3784 (9.6%)	2975 (9.0%)	806 (12.6%)	
	UCLA	14343 (36.4%)	12827 (38.9%)	1516 (23.6%)	
	UCSD	7728 (9.6%)	6067 (18.4%)	1661 (25.9%)	
	UCSF	4001 (10.2%)	3400 (10.3%)	601 (9.4%)	

© 2025 Marini S et al. *JAMA Network Open*.

IQR: interquartile range; TBI: Traumatic Brain Injury; UCD: University of California, Davis; UCI: University of California, Irvine; UCLA: University of California, Los Angeles; UCSD: University of California, San Diego; UCSF: University of California, San Francisco. *Cell sizes less than 10 are redacted in accordance with data privacy and reporting standards**.**
**Defined as Asian Pacific Islander, Hawaiian, or Middle Eastern, and included those who selected multiple races or ethnicities.

© 2025 Marini S et al. *JAMA Network Open*.

eTable 5. Baseline Characteristics of the Northwestern Medicine Cohort

Characteristic		Control group N=16,222	Mild TBI N=13,682	Moderate to Severe TBI =2,540	p-value
Sex	Female	10,190 (62.8%)	9,007 (65.8%)	1,183 (46.6%)	<0.001
	Male	6,032 (37.2%)	4,675 (34.2%)	1,357 (53.4%)	
Age at TBI, years (median)		54 (37-69)	52 (36-68)	63 (45-75)	<0.01
Race and ethnicity	American Indian or Alaska Native	<10 (<0.10%)	<10 (<0.10%)	<10 (<0.10%)	0.010
	Asian	199 (1.2%)	160 (1.2%)	39 (1.5%)	
	Black	1,451 (8.9%)	1,275 (9.3%)	176 (6.9%)	
	Hispanic	242 (1.5%)	205 (1.5%)	37 (1.5%)	
	White	14,002 (86%)	11,757 (86%)	2,245 (88%)	
	Other or unknown race or ethnicity**	328 (2.0%)	285 (2.1%)	43 (1.7%)	
Follow up in years (median, IQR)		5.8 (4-8.3)	6.3 (4.5-9)	5.7 (3.8-8.4)	0.12
Numbers of encounters after index date (IQR)		27 (11-61)	41 (16-92)	40 (16-90)	<0.001
Numbers of encounters before index date (IQR)		11 (5-26)	16 (7-40)	17 (7-42)	<0.001
Malignant Brain Tumor incidence		13 (<0.1%)	13 (<0.1%)	<10* (0.2%)	0.40

IQR: interquartile range; TBI: Traumatic Brain Injury; *Cell sizes less than 10 are redacted in accordance with data privacy and reporting standards. **Defined as Asian Pacific Islander, Hawaiian, or Middle Eastern, and included those who selected multiple races or ethnicities.

© 2025 Marini S et al. *JAMA Network Open*.
