# Supplementary material for: Traumatic Brain Injury and Risk of Malignant Brain Tumors in Civilian Populations
Source: JAMA Netw Open. 2025 Aug 25;8(8):e2528850. doi: 10.1001/jamanetworkopen.2025.28850 (PMC12379090; doi:10.1001/jamanetworkopen.2025.28850)
Supplement: Supplement 2. — Data Sharing Statement [file jamanetwopen-e2528850-s002.pdf]

Data Sharing Statement

Marini. Traumatic Brain Injury and Risk of Malignant Brain Tumors in Civilian Populations.
JAMA Netw Open. Published August 25, 2025. doi:10.1001/jamanetworkopen.2025.28850

Data
Data available: Yes
Data types: Deidentified participant data. Anonymized data not published within this article will be made available by request from any qualified investigator.
How to access data: sizzy@bwh.harvard.edu
When available: With publication

Supporting Documents
Document types: Statistical/analytic code
How to access documents: sizzy@bwh.harvard.edu
When available: With publication

Additional Information
Who can access the data: researchers whose proposed use of the data has been approved
Types of analyses: for a specified purpose as evaluated by the corresponding author
Mechanisms of data availability: after approval of a proposal
